# New insights into the micromixer with Cantor fractal obstacles through genetic algorithm

**DOI:** 10.1038/s41598-022-08144-w

**Published:** 2022-03-09

**Authors:** Xueye Chen, Honglin Lv

**Affiliations:** 1 College of Transportation, Ludong University, Yantai, 264025 Shandong China; 2 Faculty of Mechanical Engineering and Automation, Liaoning University of Technology, Jinzhou, 121001 Liaoning China

**Keywords:** Biochemistry, Biotechnology

## Abstract

This work is mainly to combine fractal principle with multi-objective genetic algorithm, and the multi-objective optimization of the Cantor fractal baffle micromixer is carried out. At different Reynolds numbers (Res), the three-dimensional Navier–Stokes equation is employed to numerically analyze the fluid flow and mixing in the microchannel. We choose the ratio of the three parameters associated with the geometry of the micromixer as design variables, and take the mixing index and pressure drop at the outlet of the micromixer as two objective functions for optimization. For the parameter study of the design space, the Latin hypercube sampling (LHS) method is used as an experimental design technique, and it is used to select design points in the design space. We use the proxy modeling of the response surface analysis (RSA) to approximate the objective function. The genetic algorithm is used to get the Pareto optimal frontier of the micromixer. K-means clustering is used to classify the optimal solution set, and we select representative design variables from it. Through multi-objective optimization, when Re = 1 and 10, the optimized mixing efficiency of the micromixer increased by 20.59% and 14.07% compared with the reference design, respectively. And we also prove that this multi-objective optimization method is applicable to any Res.

## Introduction

The microfluidic mixing system can be applied in the fields of chemical analysis, biochemical sensing, molecular separation, nucleic acid sequencing, and analytical environment monitoring^1–4^. Compared with the traditional dynamic chemical analyzer that relies on stirring and mixing, it has the characteristics of light weight, fast response and low cost. It can replace part of the existing fluid system, and the research on its mechanism and its devices has been a research hotspot in recent years. According to the presence or absence of an external driver, the micromixer can be divided into two types: active and passive^5–8^. Active micromixers use external energy sources, such as electrokinetic, ultrasonic vibration, and magnetic fields to generate flow disturbances in the microchannels^7,9–13^. However, due to the high driving voltage, the cost does not match the power of the micro device. And they are very difficult to manufacture, so they are greatly restricted in application. The passive micromixers do not require external force, but they use the geometry of the micromixer to generate a complex flow field. At present, a variety of passive micromixers such as layered, T-type and C-type have been developed. These micromixers rely on molecular diffusion to achieve fluid flow and achieve effective mixing.

In recent years, many researchers have studied different geometries to design efficient passive micromixers, such as nozzle-diffuser-like obstacle^14^, square obstacle^15^, Koch fractal obstacle^16^, spiral baffle^17^, circular section^18^ and vortex^19^. Chen and Tian^20^ designed a micromixer based on the Koch fractal structure and conducted in-depth analysis on this basis. They used the method of numerical simulation to study and analyze the factors that affect the mixing efficiency of the fluid within the microchannel. Chen et al.^21^ introduced a simple tree-shaped microchannel and discussed the fractal level of the fractal tree and the influence of the branch in the angular increment on the performance of the micromixer. Rasouli et al.^22^ combined Taguchi's experimental design and multi-objective optimization to obtain the Pareto optimal frontier of the embedded baffle-type micromixer, so that the relationship between the various objectives can be weighed. The above studies show that the fractal structure baffle in the microchannel can help to improve the mixing performance of the micromixer, and has a certain promoting effect on the development of this field. Multi-objective optimization can make it easier for us to find a micromixer with excellent mixing performance. This paper proposes for the first time the micromixer optimization method that combines the fractal principle and multi-objective optimization based on intelligent algorithms. We study the multi-objective optimization^23–26^ of the micromixer based on Cantor fractal baffle to find the micromixer with excellent mixing performance and pressure drop. It is believed that with the continuous research and exploration of experts, the combination of fractal and intelligent algorithms^27–29^ for objective optimization will definitely bring convenience to human production and life. It is foreseeable that this method will become one of the foundations of human research in a hundred years.

In this paper, we propose for the first time the combination of fractal principle and multi-objective genetic algorithm, and mainly study the multi-objective optimization of the micromixer based on the Cantor fractal principle^30^. Three-dimensional Navier–Stokes^31^ equations are used to analyze flow and mixing. The Cantor fractal principle has unique geometric properties, so we predict that this geometric property will be very useful. Multi-objective optimization has a more intelligent search for better micromixers. We choose the ratio of the three parameters as the design variables. The mixing index and pressure drop of the micromixer are used as the objective function. The RSA^32^ function is used as the proxy modeling^33–35^ of the objective function. In the case of different Res, we use the Pareto genetic algorithm^36^ to optimize the objective function, and find the Pareto optimal frontier that represents the trade-off between the objective function. The K-means clustering^37^ method is used to classify the optimal solution set to obtain representative design variables.

## Micromixer modeling and numerical analysis

### Fractal principle

**Figure 1:**
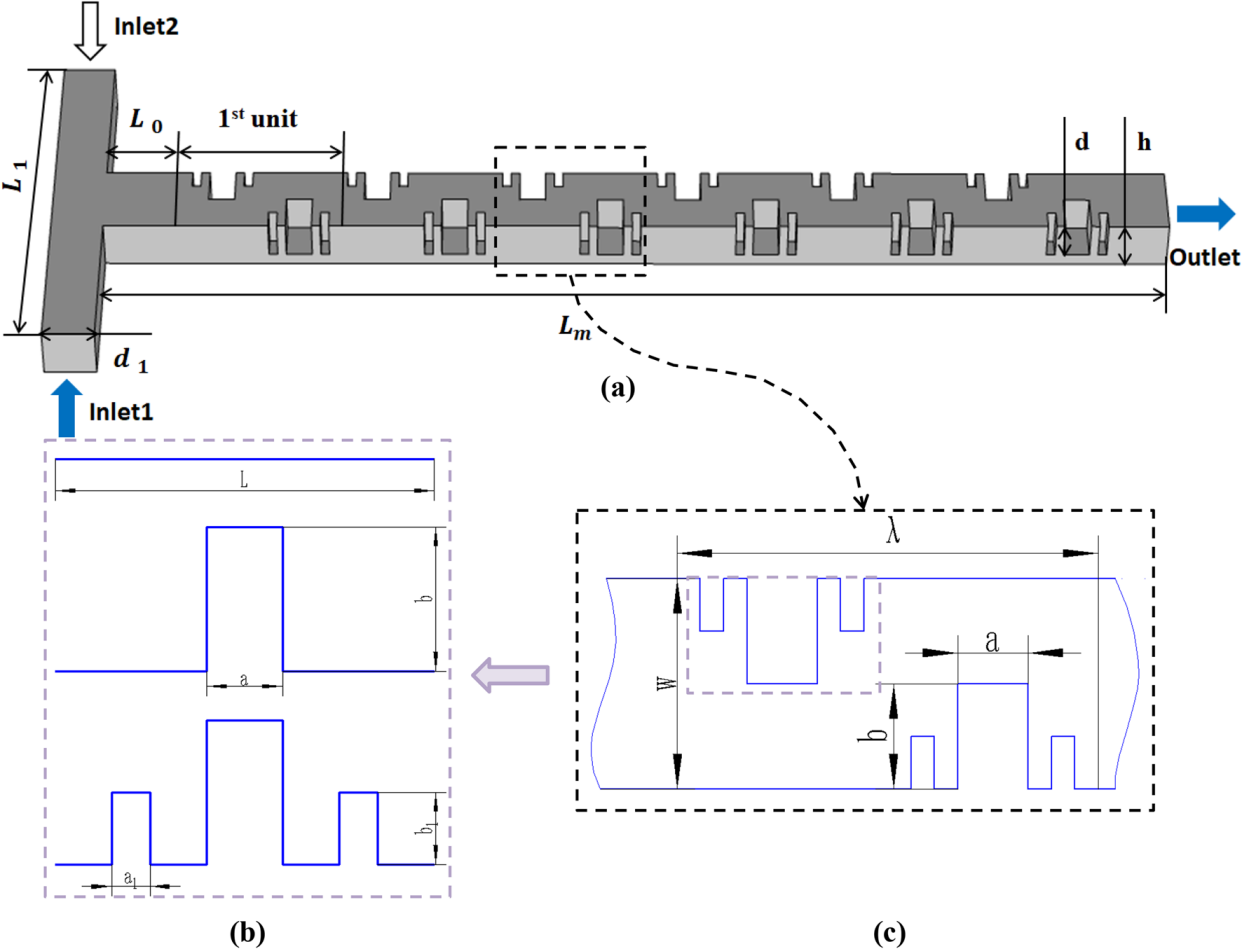
Micromixer model, (**a**) three-dimensional image of micromixer based on Cantor fractal principle, (**b**) baffle structure based on Cantor fractal principle, (**c**) design variable parameters of Cantor fractal baffle in mixing unit.

The Cantor fractal principle was first proposed by the German mathematician named Cantor. It is a collection of point sets obtained from simple straight line segments. Cantor defines this set of points in an abstract way. The most popular structure currently is the Cantor rule of thirds. It works by removing the middle part of a line segment and repeating the process on the remaining two segments. Figure 1b shows the baffle structure based on the Cantor fractal principle. A line segment of length L is divided into three equally. The initial height of the fractal barrier is a, and the length of the first-level fractal barrier is b. The length of the second-level fractal barrier is b_1_ and the height is a_1_. The theory of fractal geometry has only existed for decades, but its influence on many disciplines is extremely huge. Because the research of fractal can be applied in various fields, many scientists have also started the research and application of fractal geometry. Although the science of fractals has been born shortly, its potential use and wide range of applications are very promising. We believe that with the continuous research and exploration of experts, the applications of fractals will become more and more common in our daily life. It will definitely bring convenience to human production life.

### Micromixer modeling and numerical simulation

The micromixer channel based on Cantor fractal baffle studied in this paper has two inlets and one outlet. In order to better show the three-dimensional model of the microchannel, we use Fig. 1a to show the three-dimensional image of the micromixer with the Cantor baffle. SolidWorks software is used to design the geometry of the Cantor fractal baffle micromixer. The length of the inlet of the micromixer (L_1_) is 1.5 mm. The width (d_1_) is 0.2 mm, and the distance from the inlet to the first mixing unit (L_0_) is 0.3 mm. Each mixing unit has two baffles based on Cantor fractal in staggered arrangement. There are six mixing units in total. The length of the reference design of the mixing unit ($\lambda$) is 0.6 mm, and the height of the fractal baffle (d) is 0.15 mm. The length (a) and width (b) are 0.1 mm and 0.15 mm, respectively. The total length of the microchannel (L_m_) is 4.1 mm, and the microchannel height (h) is 0.2 mm. The above parameters for the design of the micro-mixer structure are summarized in Table 1.

**Table 1:** Parameters of the micromixer structure.

Numbers	Parameter	Description	Value (mm)
1	$L_{1}$	The length of the inlet of the micromixer	1.5
2	$d_{1}$	The width of the microchannel inlet	0.2
3	$L_{0}$	Distance from the inlet to the first mixing unit	0.3
4	$L_{m}$	The total length of the the microchannel	4.1
5	d	The height of the fractal baffle	0.15
6	a	The length of the fractal baffle	0.1
7	b	The width of the fractal baffle	0.15
8	h	The height of the microchannel	0.2
9	w	The width of the microchannel	0.3
10	$\lambda$	The length of one mixing unit	0.6

The fluid used in our numerical simulations is assumed to be a stable incompressible Newtonian fluid. Such fluid is usually set to be water. To investigate the mixing effect of the two fluids, the sample concentrations of Inlet1 and Inlet2 are set to c_1_ = 0 mol/L, c_2_ = 1 mol/L, respectively. The physical properties and constant parameters of the fluids are summarized in Table 2.

**Table 2:** Physical properties of fluid.

Numbers	Parameter	Description	Value
1	ρ	Fluid density	998 [kg/m^3^]
2	D	Diffusion coefficient of the solution	1e−11 [m^2^/s]
3	Re	Reynolds number	1, 10
4	C_1_	The concentration of sample species in Inlet1	0 [mol/L]
5	C_2_	The concentration of sample species in Inlet2	1 [mol/L]

**Figure 2:**
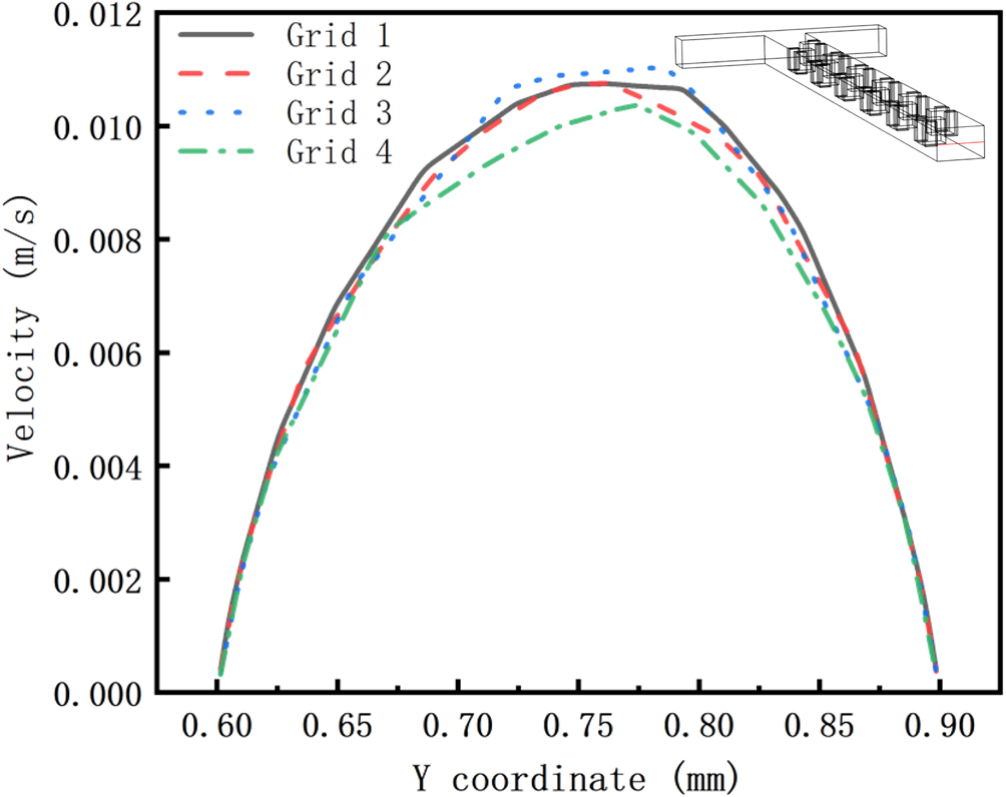
Grid independence test.

In order to determine the grid division, in this paper we have performed a grid independence test. As shown in Fig. 2, Grid 2 has been able to achieve the same results as Grid 1. Because the more the number of grides, the slower the simulation velocity is. Finally, we choose Grid 2 as the best grid. The statistics of the complete grid include 76,725 vertices, 241,986 tetrahedra, 7504 pyramids, 50,964 prisms, 32,522 triangles, 132 quadrilaterals, 3462 edge cells, 304 vertex cells and 300,454 cells.

### Control equations for species mixing

The liquid flow in the micromixer will be delineated by the Navier–Stokes equation and also the continuity equation as the following equations^8^:

1 $${\uprho }\frac{\partial u}{{\partial t}} + {\uprho }\left( {{\text{u}} \cdot \nabla } \right){\text{u}} + \nabla {\text{p}} - {\upeta }\nabla^{2} u = 0$$

2 $$\nabla \cdot {\text{u}} = 0$$

where u is the velocity vector, ρ is the fluid density, η is the dynamic viscosity and p is the pressure. Equation (1) is the equation of momentum balance. Equation (2) is the equation of continuity for incompressible fluids.

In order to study the blending within the micromixer, the convection–diffusion equation will be used. It is delineate by the subsequent Eq. (3)^8^:

3 $$\frac{\partial c}{{\partial t}} + \nabla \cdot \left( { - D\nabla c} \right) = - u\nabla c$$

where c is the concentration, D is diffusion coefficient and u is the velocity vector.

The Re of the square microchannel is calculated by the following equation^30^:

4 $$D_{d} = \frac{2HW}{{H + W}}$$

5 $${\text{Re}} = \frac{{vD_{d} }}{\gamma }$$

where Re is the Reynolds number, ${\text{D}}_{{\text{d}}}$ is the equivalent diameter, H is the height, W is the width, v is the velocity and ν is the kinematic viscosity.

In order to investigate the degree of species commixture, the variance of the commixture potency of any crosswise within the microchannel are often calculated by the subsequent Eq. (6)^30^:

6 $${\upsigma }^{2} = \frac{1}{N}\mathop \sum \limits_{i = 1}^{N} \left( {c_{i} - \overline{c}} \right)^{2}$$

where N is the total number of sampling points, ${\text{c}}_{i}$ and ${\overline{\text{c}}}$ are normalized concentration and expected normalized concentration, respectively.

The mixing index of the micromixer may be calculated by the subsequent Eq. (7)^30^:

7 $${\text{M}} = 1 - \sqrt {\frac{1}{N}\mathop \sum \limits_{i = 1}^{N} \left( {\frac{{c_{i} - \overline{c}}}{{\overline{c}}}} \right)^{2} }$$

where M is the mixing index, N is the total number of sampling points, ${\text{c}}_{i}$ and ${ }\overline{c}$ are normalized concentration and expected normalized concentration, respectively. Mixing efficiency, is that ${\text{M}} \times 100\%$, and it ranges from 0 (0%, not mixing) to 1 (100%, full mixing).

## Multi-objective optimization

### Design variables and objective functions

Since some parameters of the micromixer may have little effect on the objective function, it is important to choose the design variables reasonably. In this study, the following three design variables are selected to optimize the shape of the micromixer. As shown in Fig. 1c, we choose the ratio of the width to the length of the Cantor fractal baffle (a/b) in the mixing unit, the ratio of the width of the inlet to the length of the mixing unit (w/λ) and the height of the baffle to the height of the micromixer channel (d/h) are used as design variables. Design variables are to be determined within a reasonable range, and the design range is based on experimental research and numerical constraints. Table 3 shows these design variables and their ranges.

**Table 3:** The value range of the design variables.

Range	Design variables		
	a/b	${\text{w}}/{\uplambda }$	${\text{d}}/{\text{h}}$
Minimum (Min)	0.20	0.42	0.50
Maximum (Max)	1.00	0.80	1.00

We chose the objective function reflecting the performance of the micromixer, namely the mixing index and pressure drop. Because they have a certain importance in the function of the micromixer. The current multi-objective optimization problem can be described as^24^:

$${\text{V}} - {\text{min }}F\left( x \right) = \left[ {f_{1} \left( x \right),{ }f_{2} \left( x \right), \ldots ,f_{n} \left( x \right){ }} \right]^{T}$$

$${\text{Min}} \le {\text{x}} \le {\text{Max}},{\text{ x}} \in {\text{R}}$$

where V-min is the minimization of the vector, namely each sub-objective function in the vector objective ${f}_{i}(x)$ is minimized as much as possible, F(x) is the vector of the objective function, and x is the vector of the design variable. Min and Max are respectively the boundary value of the range of the design variable range.

We take the mixing index (M) and pressure drop (∆P) at the outlet of the micromixer as the objective function. Because multi-objective optimization is looking for a minimum value, the mixing index takes a negative value, namely $f_{1} \left( x \right) = - M$, $f_{2} \left( x \right) =\Delta P$.

We use design of experiments (DOE) to construct the design space, and use the LHS^38^ method to determine the design points within the design range. In order to obtain the LHS design points, we use the ‘lhsnorm’'^39^ function on the Matlab R2019b to select a uniform distribution of 30 for the three design variables. COMSOL Multiphysics 5.5 software is a numerical simulation software based on the finite element method. It can solve some partial differential equations of physical simulation. Table 4 lists the numerical simulation results at these design points under different Res. LHS is a sampling technique that can be used for multi-optimization. In this sampling plan, the homogeneity of the sampling plan can be maintained by measuring the maximum and minimum distances between the design points, so as to achieve uniform value everywhere in the design range.

**Table 4:** Design variables and objective function values at LHS design points for different Res.

Design points	Design variables			Objective functions			
	a/b	${\text{w}}/{\uplambda }$	${\text{d}}/{\text{h}}$	M (Re = 1)	ΔP (Re = 1)	M (Re = 10)	ΔP (Re = 10)
1	0.4039	0.5664	0.7065	0.7973	72.6341	0.8522	782.7840
2	0.7175	0.5140	0.7880	0.7241	26.9210	0.7424	297.0948
3	0.3627	0.5104	0.8490	0.9221	75.7729	0.9747	871.3308
4	0.4484	0.5808	0.7545	0.7795	36.0354	0.8421	408.6352
5	0.4029	0.4953	0.8755	0.9809	180.0933	0.9951	2048.429
6	0.2819	0.6269	0.5925	0.6179	23.6500	0.6121	242.9419
7	0.2307	0.4996	0.9545	0.9864	168.435	0.9953	2015.563
8	0.3059	0.5568	0.6340	0.7202	41.4072	0.7193	433.8979
9	0.3958	0.5961	0.5265	0.6308	23.7748	0.6317	252.1303
10	0.5186	0.5488	0.9220	0.8923	46.1576	0.9307	528.6534
11	0.5142	0.5418	0.7355	0.7987	43.0451	0.8711	483.9600
12	0.3424	0.6487	0.8465	0.9280	73.7338	0.9815	841.1057
13	0.6394	0.6127	0.7195	0.5388	13.5311	0.5136	143.9363
14	0.3261	0.6284	0.6255	0.6382	22.8918	0.6647	250.2992
15	0.2748	0.6086	0.3835	0.5944	20.0948	0.5818	206.3002
16	0.5099	0.4812	0.8960	0.9295	72.8003	0.9608	832.9332
17	0.5645	0.4138	0.6885	0.8270	49.7706	0.8649	541.6068
18	0.3855	0.7199	0.7410	0.6934	29.0481	0.8052	330.6337
19	0.3502	0.6747	0.8225	0.7293	26.2941	0.8046	312.4337
20	0.3723	0.6326	0.5665	0.5227	14.9721	0.4971	159.2088
21	0.5548	0.5150	0.6595	0.7149	31.2719	0.7695	343.7368
22	0.3226	0.4937	0.8500	0.9538	125.5217	0.9878	1433.105
23	0.8347	0.6091	0.8030	0.5509	13.8853	0.5170	146.0607
24	0.4847	0.7829	0.5880	0.5580	21.8651	0.6113	236.7469
25	0.4663	0.5227	0.4965	0.6937	29.2291	0.6917	305.9860
26	0.7093	0.6061	0.6850	0.6742	24.5116	0.7243	271.2799
27	0.1852	0.5810	0.7935	0.9078	93.0807	0.9148	1005.118
28	0.5991	0.4244	0.9245	0.8976	66.2350	0.9315	736.7491
29	0.4049	0.6387	0.7750	0.8301	56.2694	0.9254	626.5036
30	0.2769	0.5572	0.5000	0.6323	35.4253	0.6403	365.3808

The mathematical method of RSA is used to establish a proxy modeling for the above design points. Finally, a multi-objective optimization algorithm is used to optimize the design variables within the design range, so as to obtain the Pareto optimal frontier solution set. We randomly selected a set of reference designs. Table 5 lists the numerical simulation results of the reference designs under different Res.

**Table 5:** Design variables of the reference design and its objective function value for different Res.

Reference design						
Design variables			Objective functions			
a/b	${\text{w}}/{\uplambda }$	${\text{d}}/{\text{h}}$	M (Re = 1)	ΔP (Re = 1)	M (Re = 10)	ΔP (Re = 10)
0.67	0.50	0.75	0.7886	37.3961	0.8431	413.1535

### Response surface function and proxy modeling

Proxy modeling of the objective function is used to solve the calculation problem of multi-objective optimization. This paper uses RSA method to approximate the objective function value in various proxy modeling techniques. In Matlab, the ‘regstats’^39^ function is employed to calculate the operate of RSA design. In the RSA operation technology, the continuous response y is usually carved as a linear combination of the operation of the programming variable x and the random error ε:

8 $${\text{y}} = \beta_{1} f_{1} \left( x \right) + \beta_{2} f_{2} \left( x \right) + \cdots + \beta_{m} f_{m} \left( x \right) + \varepsilon$$

where x is a vector of design variables, the model term is ${\text{f}}_{{\text{i}}}$(x) (i = 1……m), the coefficient is ${\upbeta }_{i}$ (i = 1……m), the error ε is assumed to be uncorrelated, and its mean value is 0. The variance is an unknown constant^32^. For N independent observations of M design variables, the model can use ${\text{N}} \times {\text{m}}$ equation group describe it, namely ([${\text{x}}_{11}$ ${\text{x}}_{12}$…${\text{x}}_{{1{\text{m}}}}$], ${\text{y}}_{1}$), …, ([${\text{x}}_{{{\text{N}}1}}$ ${\text{x}}_{{{\text{N}}2}}$…${\text{x}}_{{{\text{Nm}}}} ], {\text{y}}_{{\text{N}}} )$. In the matrix form, the system can be expressed as^32^:

9 $$\left( {\begin{array}{*{20}c} {\begin{array}{*{20}c} {y_{1} } \\ . \\ . \\ . \\ \end{array} } \\ {y_{n} } \\ \end{array} } \right) = \left( {\begin{array}{*{20}c} {f_{1} \left( {x_{1} } \right)} & \cdots & {f_{m} \left( {x_{1} } \right)} \\ \vdots & \ddots & \vdots \\ {f_{1} \left( {x_{N} } \right)} & \cdots & {f_{m} \left( {x_{N} } \right)} \\ \end{array} } \right)\left( {\begin{array}{*{20}c} {\beta_{1} } \\ {\begin{array}{*{20}c} . \\ . \\ {\beta_{m} } \\ \end{array} } \\ \end{array} } \right) + \left( {\begin{array}{*{20}c} {\varepsilon_{1} } \\ {\begin{array}{*{20}c} . \\ . \\ {\varepsilon_{N} } \\ \end{array} } \\ \end{array} } \right)$$

where X is the design matrix of the system. The X column is the item evaluated by the model in the design variables. For objective function approximation, the coefficient ${\upbeta }_{i}$(i = 1……m) needs to be determined. The smallest amount square estimate of β is^32^:

10 $${\upbeta } = \left( {X^{T} X} \right)^{ - 1} X^{T} y$$

The constructed second-order polynomial response surface model can be expressed as^32^:

11 $${\text{y}}\left( {\text{x}} \right) = \beta_{0} + \mathop \sum \limits_{i = 1}^{N} \beta_{i} X_{i} + \mathop \sum \limits_{i = 1}^{N} \beta_{ii} X_{i}^{2} + \mathop \sum \limits_{i < j}^{N} \beta_{ij} X_{i} X_{j}$$

The right side of the Eq. (11) represents the intercept, linear term, quadratic interaction term, and square term from left to right. In order to determine the goodness of fit, we use $R^{2}$. In order to get a good match, the adjusted value of $R^{2}$ must be close to 1. In this work, the generated polynomial response surface function is treated as the proxy modeling.

### Pareto genetic algorithm

The Pareto distribution^40^ is named after Vifredo Pareto who is the Italian economist. It is a power law distribution discovered from a large number of real world phenomena. In the Pareto distribution, if X is a random variable, the probability distribution of X is shown in the following equation^40^:

12 $${\text{P}}\left( {{\text{X}} > {\text{x}}} \right) = \left( {\frac{x}{{x_{min} }}} \right)^{k}$$

where x is any range larger than ${\text{x}}_{{{\text{min}}}}$, ${\text{x}}_{{{\text{min}}}}$ is the smallest possible value (positive number) of X, and k is a positive parameter. The family of Pareto distribution curves is parameterized by two quantities: ${\mathrm{x}}_{\mathrm{min}}$ and k. The distribution density is shown by the following formula^40^:

13 $${\text{p}}\left( {\text{x}} \right) = \left\{ {\begin{array}{*{20}l} {0,} \hfill & {if\; {\text{x}} < {\text{x}}_{min} } \hfill \\ {\frac{{{\text{k}} \times x_{min}^{k} }}{{x^{k + 1} }},} \hfill & {if\; {\text{x}} > {\text{x}}_{min} } \hfill \\ \end{array} } \right.$$

The Pareto distribution is a continuous probability distribution. The expected value of a random variable that obeys the Pareto distribution is $\frac{{x_{min} \times k}}{k - 1}$ (if k ≤ 1, the expected value is infinite) and the standard deviation of the random variable is $\frac{{x_{min} }}{k - 1}\sqrt {\frac{k}{k - 2}}$ (if k ≤ 2 standard deviation does not exist). Pareto optimal solution is also known as Pareto efficiency. It refers to an ideal state of resource allocation. It is based on the assumption that there is a fixed population and allocable resources that make at least one person better off and no one worse off when moving from one allocation state to another. Pareto optimal state means that there can be no more room for Pareto improvement.

We can use the ‘gamultiobj’^39^ function that comes with Matlab to perform multi-objective optimization. The Pareto genetic algorithm describes an initial population that begins to evolve through chromosomal variation or crossover transformation, eventually resulting in the most adaptive population. Before the search starts, a series of chromosomes are randomly selected from the design space to obtain the initial population. According to the fitness function, individuals are selected in a competitive manner. We use genetic search operators (selection, mutation, crossover) to obtain the next generation of chromosomes, and all chromosomes are predicted to be of better quality than the previous generation. This process is then repeated until the functional tolerances are met. We set the following parameters: population size is 500, cross score is 0.3, generation is 600, and functional fault tolerance is 1E-10.

## Results and discussion

### The influence of design variables on the objective function

**Figure 3:**
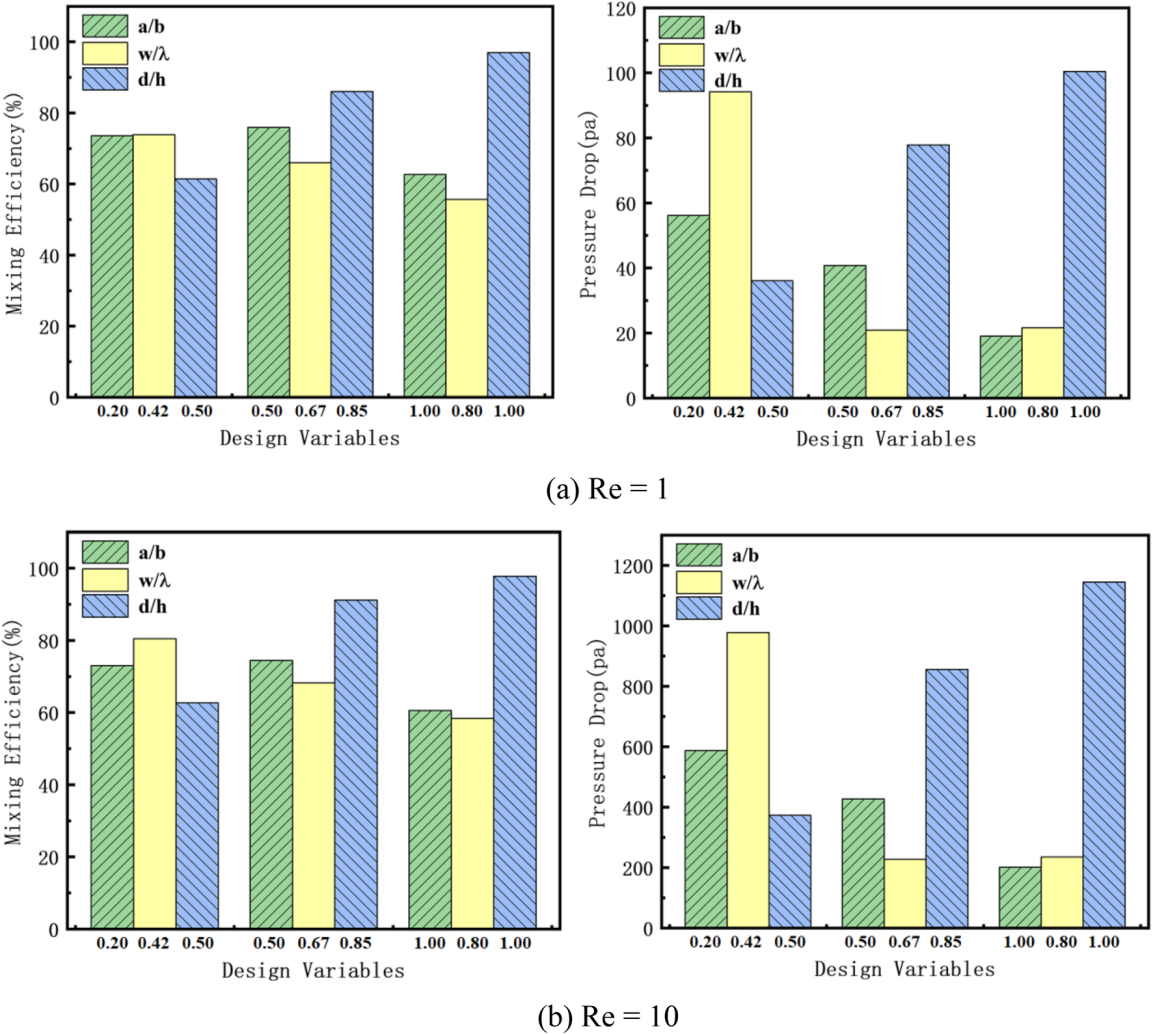
Influence of design variables on objective functions at different Res.

First, we discuss the effects of the three design variables on the mixing efficiency and pressure drop of the mixer at different Res. Three sets of design variables are selected for simulation, which are the minimum, intermediate and maximum values within the range of the design variables. It can be seen from Fig. 3 that when Re = 1 and 10, d/h has the most significant impact on the mixing efficiency, while w/λ has the most prominent impact on the pressure drop. For the mixing efficiency, a/b obtains the optimal value at the intermediate value, while the other two design variables obtain the optimal value at the boundary. For the pressure drop, the three design variables all achieve the optimal value at the intermediate value. But when the mixing efficiency achieves the optimal value, the pressure drop is the worst value. This shows that we can't find the micromixer with the best comprehensive performance by only relying on experimental analysis. So we use Pareto genetic algorithm to optimize micromixer is a good choice. It can more intelligently weigh the compromise value of the two performances, thereby obtaining a micromixer with better comprehensive performance.

### Influence of mixing unit on mixing efficiency

**Figure 4:**
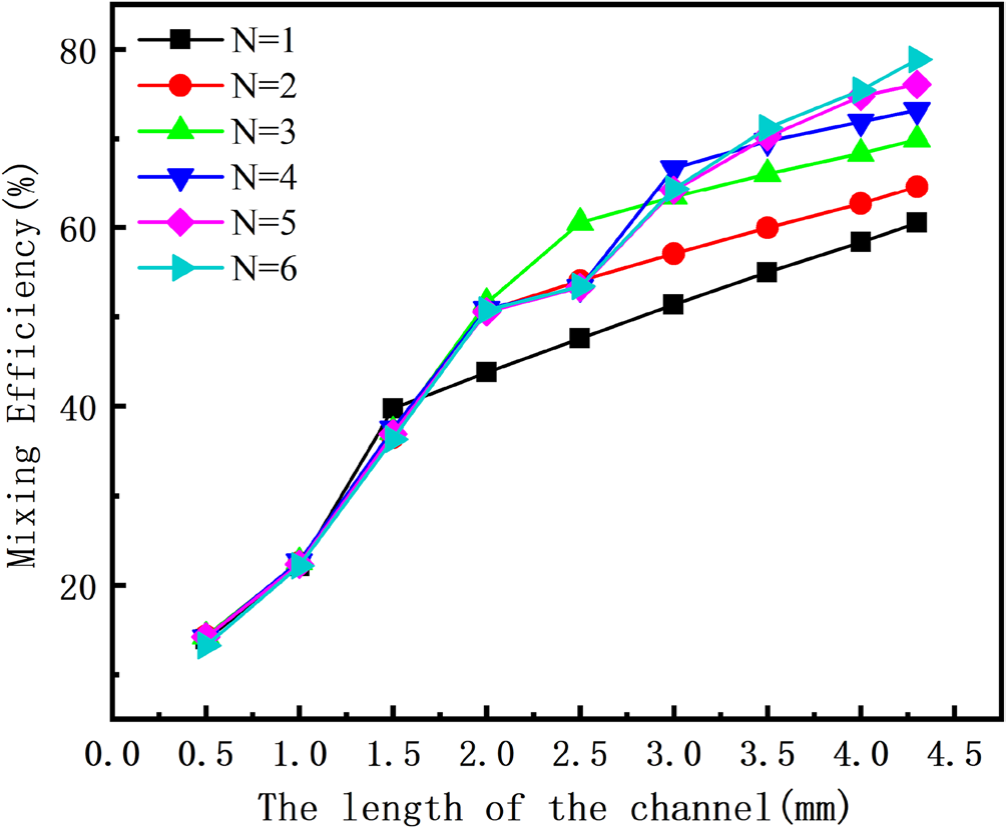
The influence of the number of mixing units on mixing efficiency.

Next, we discuss the influence of the number of mixing units on the mixing efficiency. We perform numerical simulations on the reference design micromixers with different numbers of mixing units to show the influence of the number of mixing units on the mixing performance. It can be seen from Fig. 4 that as the number of mixing units increases, the mixing efficiency at different Res increases as the length of the micromixer increases. When the number of mixing units and the length of the microchannel increase, the diffusion time and the contact area between the fluids increase. This leads to uniform mixing and improves mixing efficiency. When the number of mixing units is six, the mixing efficiency of the micromixer can reach 80%. When we select the number of mixing units and then modify the design variables within the range, it will have a fluctuating influence on the mixing efficiency. Therefore, six mixing units are selected to leave room for the fluctuation of mixing efficiency and to ensure that the optimization effect is excellent. Therefore, the six mixing units are selected to design the micromixer.

### Optimization results at different Res

**Figure 5:**
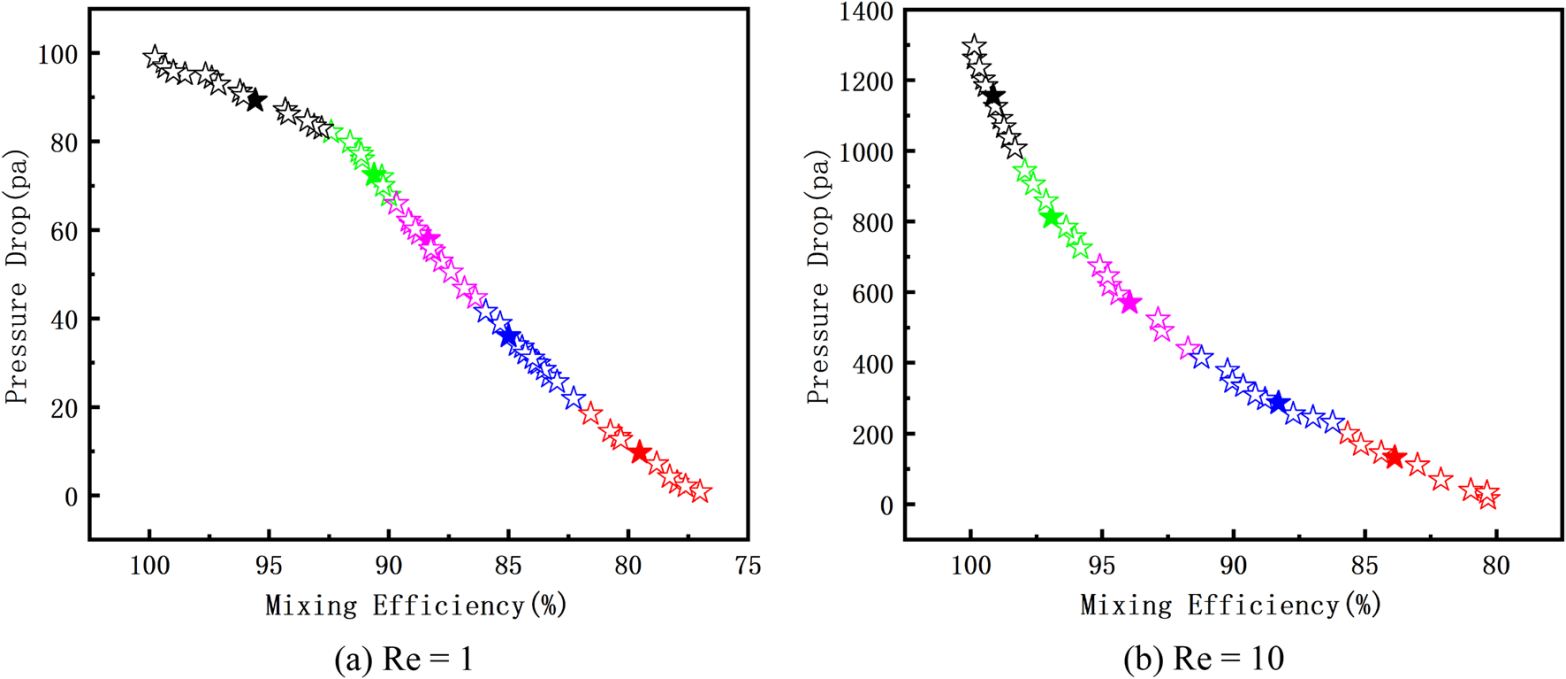
The relationship between mixing efficiency and pressure drop on Pareto optimal frontier at different Res.

From the above discussion, it can be seen that the mixing efficiency and pressure drop increase with increasing channel length when Re = 1 and 10. Therefore, finding a micromixer that combines high mixing efficiency and low pressure drop is the problem we need to investigate. We use the Pareto genetic algorithm to perform multi-objective optimization of the two objective functions of mixing efficiency and pressure drop. In order to analyze the Pareto optimal solution, we choose five representative Pareto optimal design at each Pareto optimal frontier through K-means clustering. The optimized results obtained are shown in Fig. 5. It can be seen that on OPT-A (Re = 1), the range of mixing efficiency of Pareto optimal frontier obtained by optimization is 75%-100%, and the pressure drop is in the range of 0–100 Pa. Among them, on OPT-B (Re = 10), the range of mixing efficiency of the Pareto optimal frontier is 80%-100%, and the pressure drop ranges between 0–1400 Pa.

Five representative OPT results from numerical simulations on OPT-A and OPT-B are listed in Tables 6 and 7, respectively. It is observed that the optimized design variables a/b, w/λ, and d/h are respectively close to the middle, lower and upper limits of the range. Comparing the numerical results of the two tables can show that on OPT-A, compared with the reference design (RD), the mixing efficiency of OPT-1 at the outlet is increased by 20.59%, and the pressure drop is also increased by 223 Pa. On OPT-B, the mixing efficiency at the outlet of OPT-1 is increased by 14.07% and the pressure drop is also increased by 998.75 Pa compared to RD. The mixing efficiency at the outlet of OPT-5 is increased by 5.55% and the pressure drop is also increased by 72.75 Pa. Through analysis and discussion, compared with the RD, when Re = 1 and 10, the mixing efficiency is increased by 20.59% (OPT-1 on OPT-A) and 14.07% (OPT-1 on OPT-B), respectively. In summary, the optimized results are indeed much better than the reference design. We can also make a good trade-off between the two performances of the micromixer in the optimized solution set, so that we can choose the micromixer with higher comprehensive performance.

**Table 6:** The optimization result of the representative Pareto optimization design at OPT-A.

Optimization	Design variables			Objective functions	
	a/b	${\text{w}}/{\uplambda }$	${\text{d}}/{\text{h}}$	M (Re = 1)	$\Delta$P (Re = 1)
OPT-1	0.3439	0.4975	0.9998	0.9945	260.426
OPT-2	0.5266	0.4467	0.9999	0.9269	66.0687
OPT-3	0.5748	0.4653	1.0000	0.8735	45.6592
OPT-4	0.5739	0.4935	0.9996	0.8363	37.1408
OPT-5	0.6084	0.4940	0.9998	0.7975	31.4788

**Table 7:** The optimization result of the representative Pareto optimization design at OPT-B.

Optimization	Design variables			Objective functions	
	a/b	${\text{w}}/{\uplambda }$	${\text{d}}/{\text{h}}$	M (Re = 10)	$\Delta$ P (Re = 10)
OPT-1	0.3046	0.4178	0.8803	0.9838	1411.9062
OPT-2	0.3308	0.4174	0.8624	0.9757	1006.0909
OPT-3	0.3590	0.4177	0.8707	0.9461	697.1103
OPT-4	0.3940	0.4280	0.8711	0.9305	606.2954
OPT-5	0.3957	0.4561	0.8757	0.8986	485.9058

### Mixing efficiency and pressure drop of OPTs and RD

**Figure 6:**
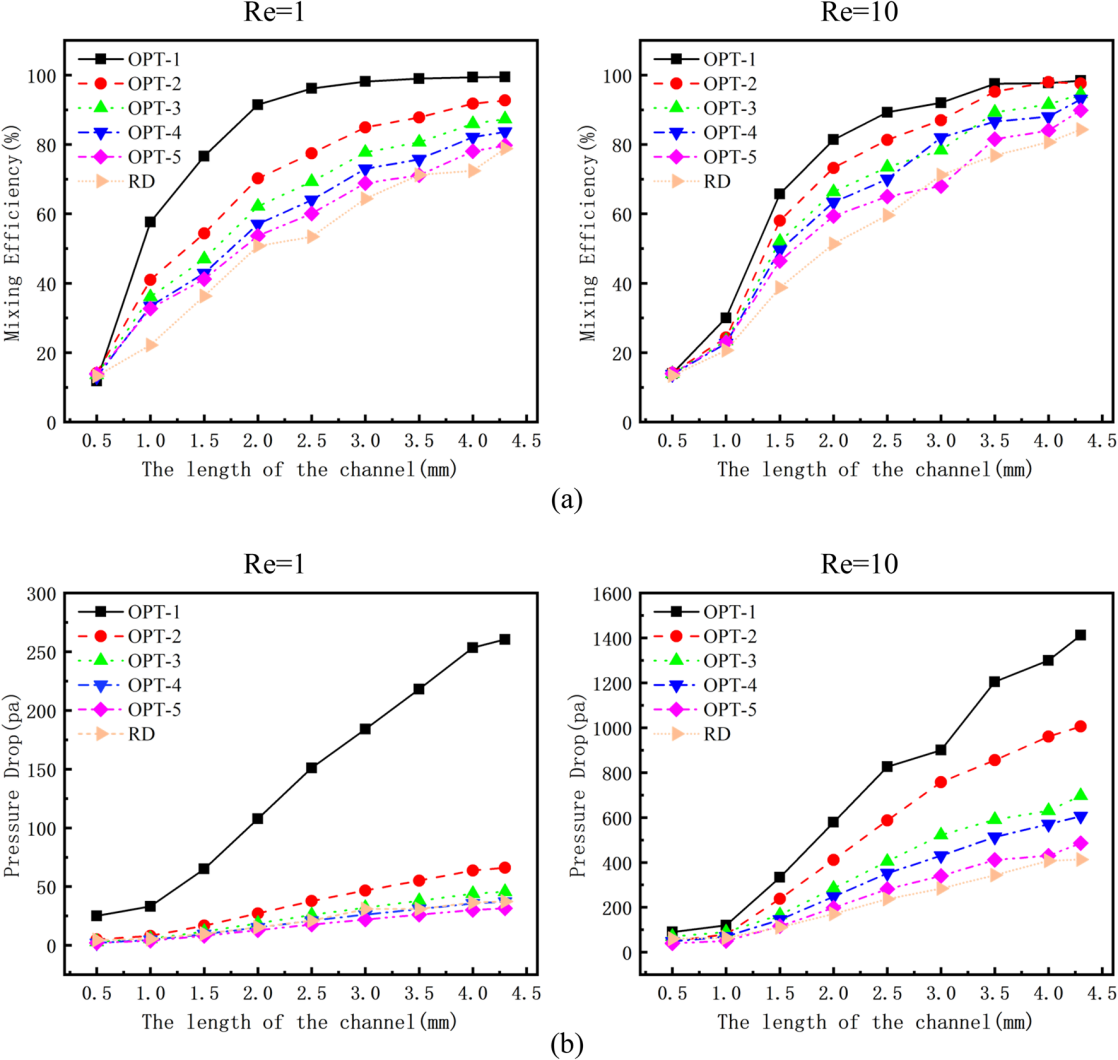
Mixing efficiency and pressure drop of OPTs and RD, (**a**) the mixing efficiency of OPT and RD varies with the length of the channel at different Res, (**b**) the pressure drop of OPT and RD varies with the length of the channel at different Res.

The micromixer optimized by the Pareto genetic algorithm forms an optimal solution set and the K-means clustering algorithm is used to divide the Pareto optimal solution set into five categories. We select five representative design variables. Figure 6a shows the mixing efficiency of the five optimized micromixers at OPT-A and OPT-B obtained by numerical simulation, and the optimized micromixer is compared with the RD. A comparison of the mixing efficiencies of the above six micromixers at different Res shows that the optimized micromixer has a slightly lower mixing efficiency than the RD at the mixing inlet. As the mixing length increases, the mixing efficiency of the optimized micromixer is gradually greater than that of the RD and the mixing efficiency of the optimized micromixer is greater than that of the RD. On OPT-A, the mixing efficiency of OPT-1 closes to complete mixing. The mixing efficiency of OPT-2 is greater than 90%. The mixing efficiency of OPT-4 is slightly lower than that of OPT-3, and the mixing efficiency of these two micromixers are both greater than 80%. Because of the optimized internal structure of the channel, the molecular diffusion capacity is increased and a large amount of chaotic convection is generated. As a result, the comprehensive performance of the micromixer is significantly improved. On OPT-B, the mixing efficiency of OPT-1 and OPT-2 are both close to complete mixing. The mixing efficiency of OPT-3 and OPT-4 is slightly lower than the first two but still greater than 90%. The mixing efficiency of OPT-5 and RD are slightly lower than 90%. Therefore, it can be concluded that when Re increases further, the flow rate of the fluid will increase and strong convection will be formed in the microchannel, so the mixing efficiency of the micromixer begins to increase.

The pressure drop is additionally one among the vital indicators for testing the performance of the micromixer. The pressure drop is that the pressure distinction between the inlet and outlet of the micromixer. The higher pressure drop in the micromixer represents the higher energy required at the inlet, resulting in a lower safety of the micromixer. Figure 6b shows the pressure drop of the optimized micromixer and RD. It can be seen that as the length of the micromixer increases, the pressure drop also increases. On OPT-A, the pressure drop of OPT-1 is the highest, which is much higher than the pressure drop of the other five micromixers. The pressure drops of the other five micromixers do not differ much from each other and are between 0 and 50 Pa. On OPT-B, the pressure drop of OPT-1 is the largest, which exceeds 1200 Pa. The pressure drop of the OPT-2 is much lower than that of the OPT-1, which is around 1000 Pa. The pressure drop of the other four micromixers are is not very different and is in the range of 400–700 Pa. When Re increases, the increased flow velocity of the two fluids in the micromixer creates a strong chaotic convection, therefore the pressure drop will increase.

### Analysis of the concentration field of the optimized micromixer

**Figure 7:**
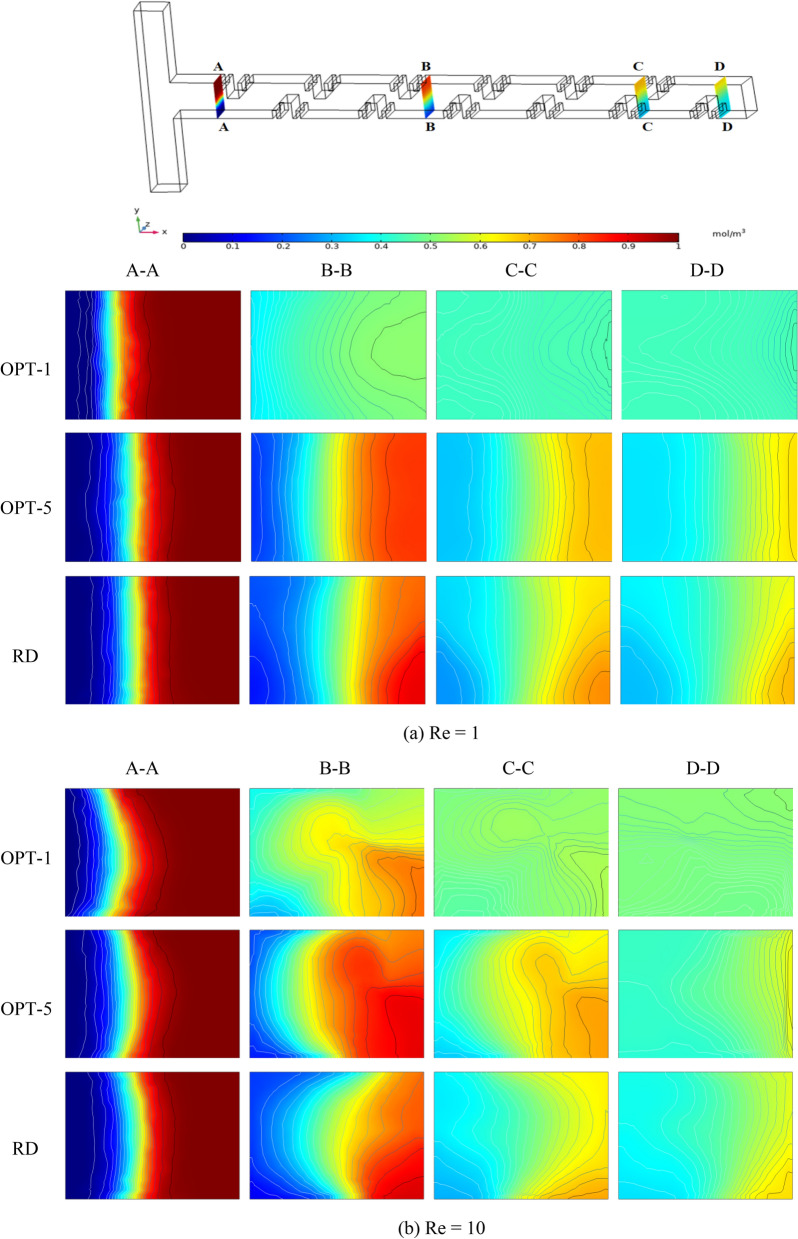
The concentration contours of OPT and RD at different Res.

In order to study the concentration distribution of the micromixer, we intercept the concentration profiles of four important cross-sections. They are the inlet of the micromixer (A-A section), the two important locations of the continuous mixing unit through the fractal structure (B-B and C-C sections), and the outlet of the micromixer (D-D section). We select two Pareto-optimal designs, OPT-1 and OPT-5, at both ends of the Pareto-optimal boundary and use them to compare with RD. By observing the Fig. 7, it can be seen that the contact area caused by the obstruction of the two fluids through the Cantor fractal baffle increases with the length of the channel. However, the distribution of the concentration contours at the outlet of the OPT-1, OPT-5 and RD on OPT-A is significantly different. Compared with OPT-1 on OPT-A, OPT-5 and RD have more layered interfaces in the concentration profile of the two fluids at section B-B, and better mixing has not been achieved. In the C-C section, the concentration of the fluid on OPT-1 has a single color and is almost completely mixed, while the concentration of both OPT-5 and RD gradually spreads and the concentration color is closer to the middle value. By the time the fluid reaches the outlet, OPT-1 has achieved perfect mixing, while OPT-5 and RD have a reduced number of density profile interfaces and fewer color types. Compared to OPT-A, the concentration distribution of the three micromixers on OPT-B is more inhomogeneous. They both exhibit vortex and spiral phenomena that improve the mixing velocity and mixing performance of both fluids. The increase of Re causes the fluid in the microchannel to produce chaotic convection, and causes the fluids of different concentrations to produce large-scale oscillations, which is conducive to the rapid mixing of the fluids. These factors greatly improve the mixing efficiency of the micromixer.

### Analysis of the velocity field of the optimized micromixer

**Figure 8:**
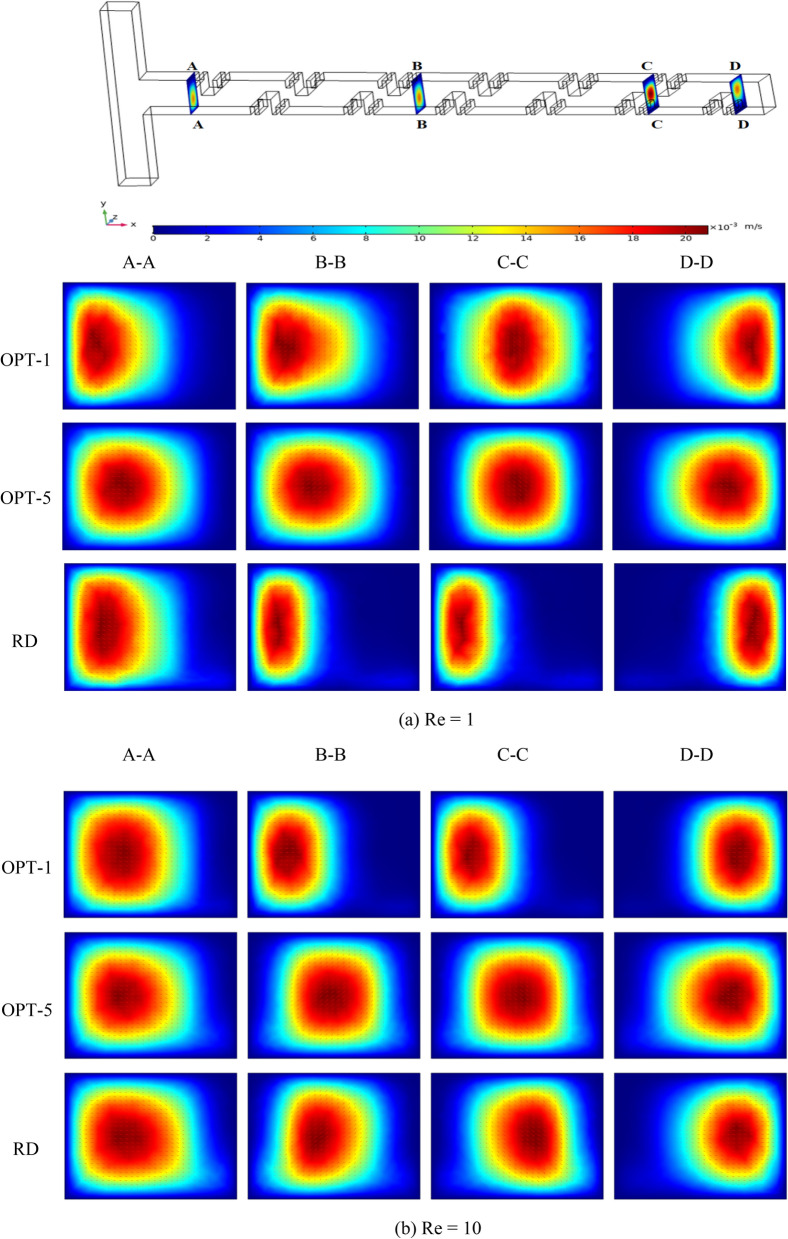
The velocity vector diagram of OPT and RD at different Res.

In order to analyze the mixing mechanism, we chose two Pareto optimal designs, namely OPT-1 and OPT-5, at both ends of the Pareto optimal frontier and compared them with RD to compare the velocity fields. Figure 8 shows the variation of the velocity vectors in the microchannel for four different cross sections. At section A-A, the sample fluids of the three types of micromixers have just arrived at the inlet of the microchannel. In the y–z plane, the fluid velocity is faster on the left side of the microchannel. In sections B-B and C-C, after the sample fluid flows through the mixing units with Cantor fractal baffle, the velocity of the fluid gradually changes to the right side of the microchannel. At the section D-D, the velocity direction of the fluid has completely changed from the left side of the microchannel to the right side of the microchannel. The results show that the fractal baffle plays an important role in changing the direction of fluid flow and enhancing convection. Compared to OPT-A, the three micromixers on OPT-B have a more intense fluid flow and a greater variation in flow direction. The fluids produce a wide range of oscillations and have better mixing performance. Finally, it is found that whether it is on OPT-A or OPT-B, the degree of change of the fluid velocity direction in OPT-1 and OPT-2 is greater than that of RD, and both produce more chaotic convection than RD. Therefore, the mixing performance of the optimized micromixer can be better than that of RD.

### The concentration surface of the micromixer is at different Res

**Figure 9:**
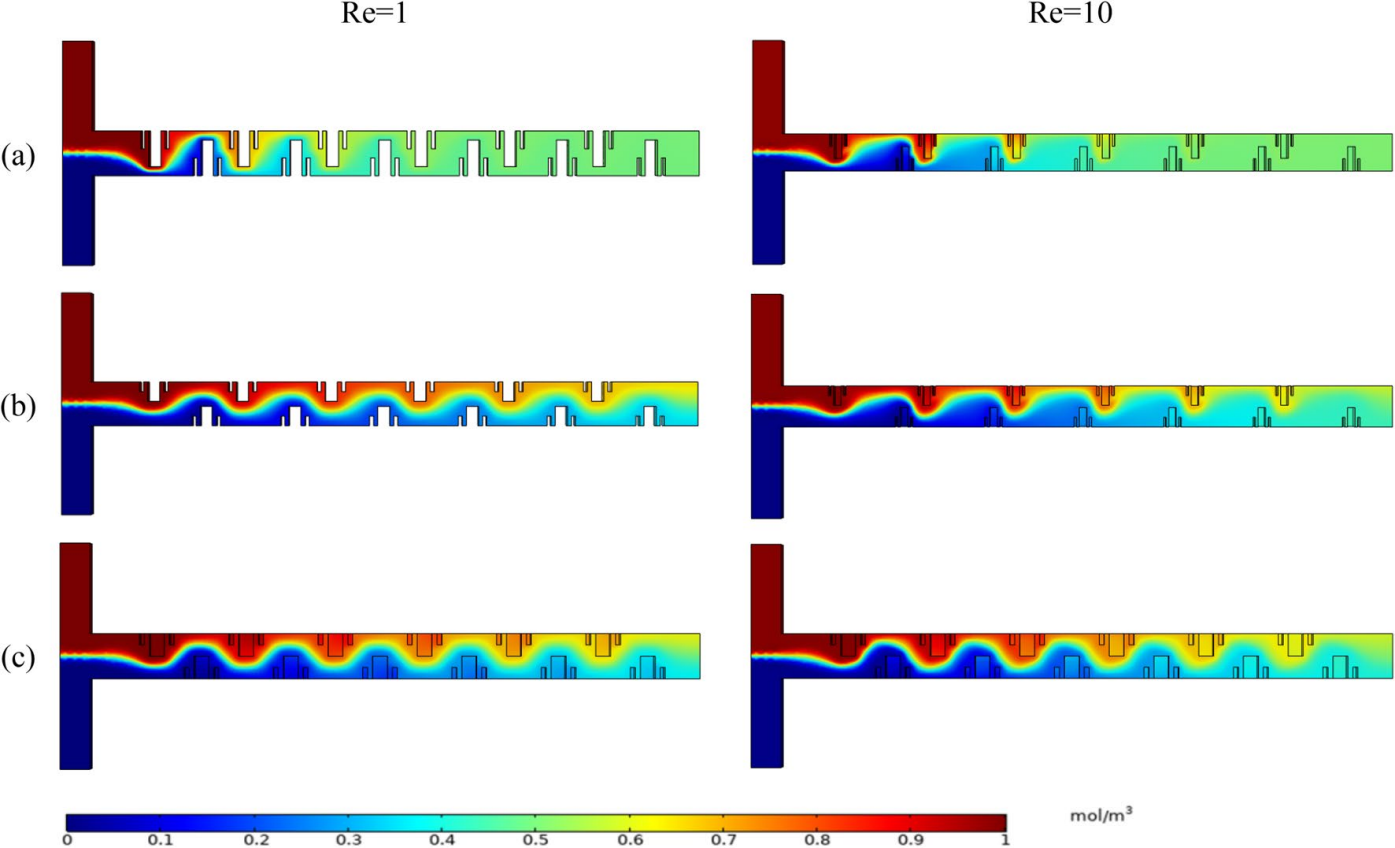
Mixing performance of OPT and RD at different Res, (**a**) OPT-1, (**b**) OPT-5, (**c**) RD.

In order to investigate more clearly the effect of Cantor fractal baffle parameters on the mixing performance of the micromixer, OPT-1, OPT-5 and RD are selected for comparison. Figure 9 shows the concentration surface of the three types of micromixers at different Res. When the fluid just enters the microchannel, the two different fluids present a symmetrical distribution in the microchannel. Once the fluid enters the mixing zone through the Cantor baffle, the two fluids change considerably within the microchannel. At the same time, the concentration of the fluid starts to change in a wavy pattern and is distributed at the top and bottom of the mixing zone on both sides. This phenomenon helps to expand the contact area between the two fluids in the microchannel and increases the molecular diffusion capacity.

On the OPT-A, the OPT-1 has the fastest mixing velocity. As the fluid flows through the four fractal baffles, the fluid is almost completely mixed. When the fluid reaches the outlet, the two fluids are perfectly mixed. The mixing performance of OPT-5 is better than that of OPT-1. Due to the parametric design of the fractal baffle, the OPT-5 achieves basic mixing at the outlet of the channel. Finally, the mixing performance of the RD is far inferior to that of the optimized micromixer. On OPT-B, OPT-1 achieves perfect mixing at the outlet of the channel, and both OPT-1 and OPT-5 can outperform RD in mixing. When Re increases, it helps to drive more chaotic convection between the two fluids, which in turn increases the mixing efficiency. The stronger rotation of the velocity streamline near the baffle makes the mixing performance better. Through the above study, it is found that the mixing performance of the micromixer optimized by Pareto genetic algorithm is better than that of RD.

### The concentration cut line of the micromixer is at different Res

**Figure 10:**
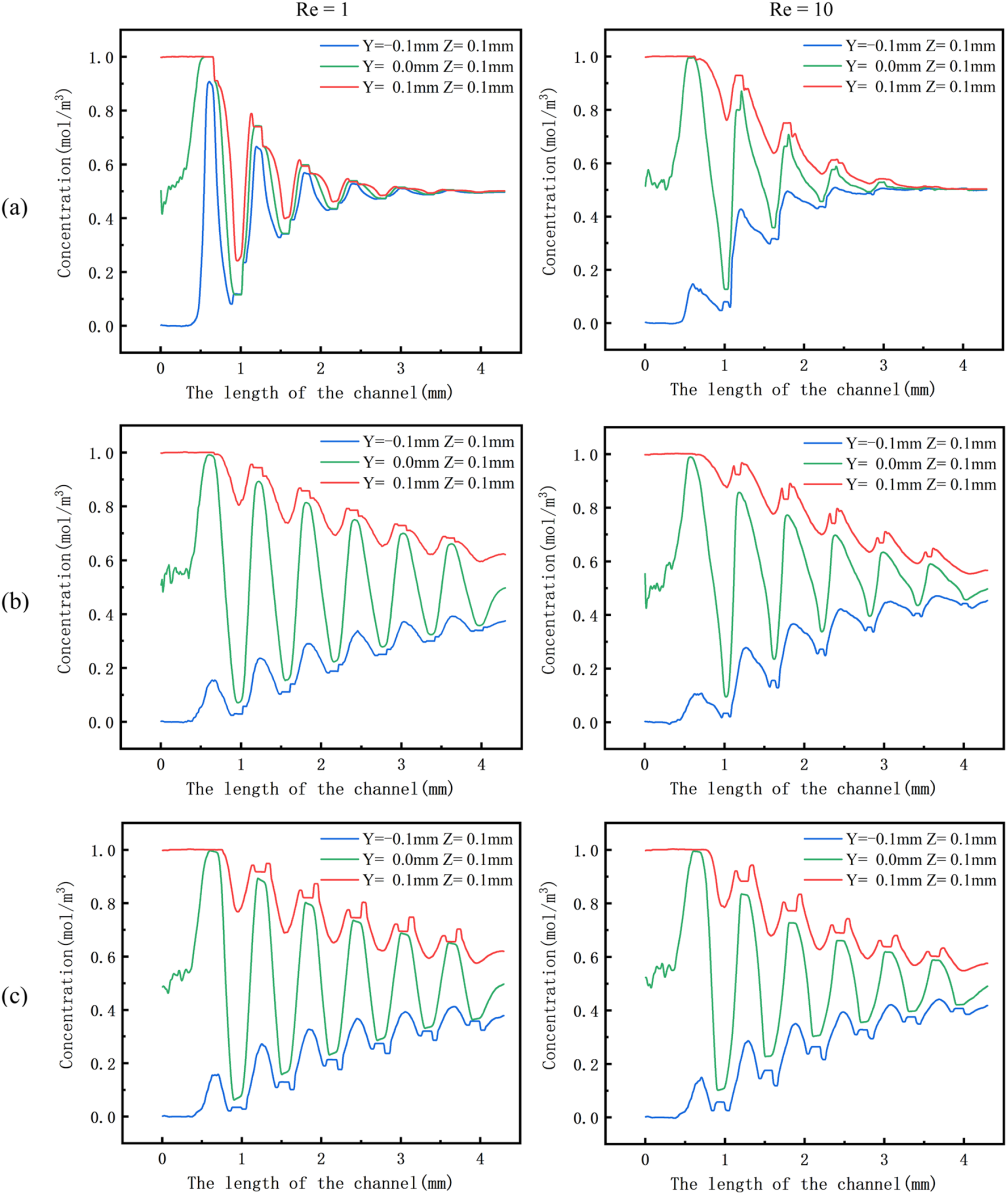
Concentration curves of OPT and RD along the length of the microchannel at different Res, (**a**) OPT-1, (**b**) OPT-5, (**c**) RD.

We study the concentration curve of the micromixer along the length of the channel, and discuss the influence of the Cantor fractal baffle on the concentration distribution of the micromixer. We chose OPT-1, OPT-5 and RD for research. On the y–z coordinate system, we collect the concentration data in the microchannel. The coordinate origin (0, 0) of the inlet of the left channel is set as the center. We take three cross-sections in the microchannel. Their coordinates on the Y-axis are 0.1 mm, 0.0 mm, and − 0.1 mm, respectively, and their coordinates on the Z-axis are all 0.1 mm. The Fig. 10 shows the concentration convergence curve at different positions at different Res. It can be seen that the X-axis concentration fluctuates greatly due to the influence of the Cantor fractal baffle.

On OPT-A, the concentration of OPT-1 at the inlet remains constant and there is little mixing. As the length of the channel increases, the two fluids begin to mix and the concentration curve begins to fluctuate dramatically. When it reaches the outlet of the micromixer, it is essentially perfect and the concentration is close to 0.5 mol/m^3^ at this moment. On OPT-5, the two fluids begin to mix as the length of the channel increases. The concentration curve fluctuation is not as severe as OPT-1 and the frequency is also low. When they reach the outlet of the micromixer, the mixing of the two fluids is not completed. The concentration does not reach 0.5 mol/m^3^. However, as the length of the channel increases, the concentration curve of the two fluids gradually approaches 0.5 mol/m^3^, but the concentration is relatively far from complete mixing when they reach the outlet.

On OPT-B, as the length of the channel increases, the fluid in OPT-1 gradually mixes and the concentration curve approaches 0.5 mol/m^3^ at the outlet. However, the fluctuations in the concentration curve are not as severe as at low Re. The fluid in OPT-5 oscillates in the channel under the influence of the Cantor fractal baffle. Because of the short residence time of the two fluids in the microchannel and the incomplete mixing of the fluids, the three concentration curves do not converge completely to 0.5 mol/m^3^ at the outlet of the micromixer. From the above analysis, it can be seen that the chaotic convection caused by the fractal baffle to the fluid flow has far-reaching significance for improving the mixing efficiency.

### Application of multi-objective genetic algorithm in different Res

**Figure 11:**
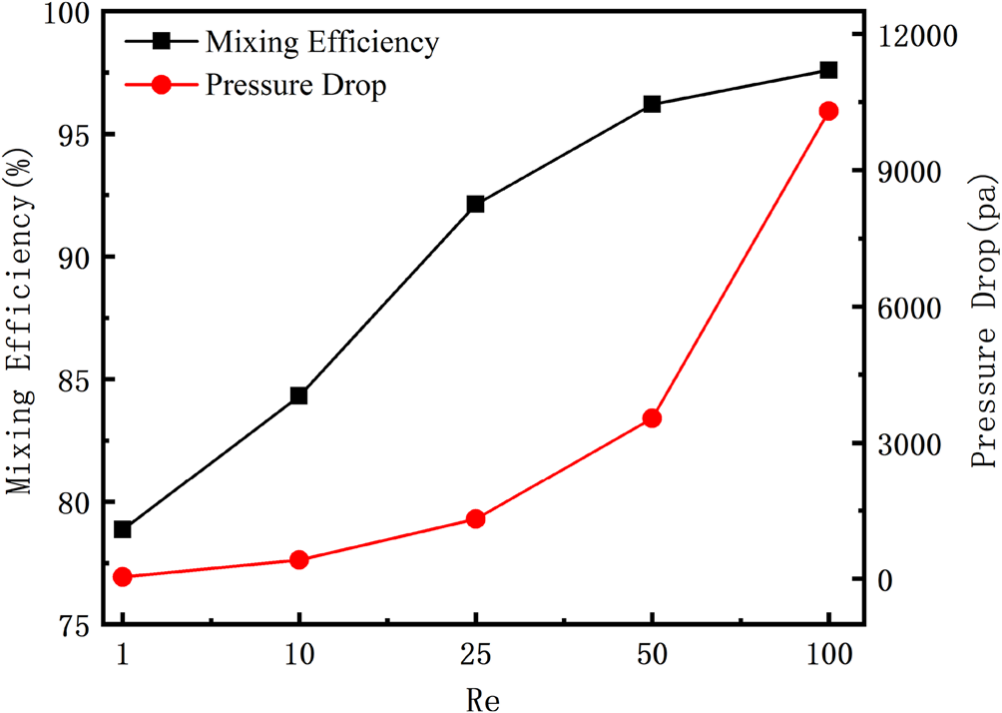
Mixing efficiency and pressure drop of RD at different Res.

In order to demonstrate the applicability of the Pareto genetic algorithm for multi-objective optimization at different Res, we select RD to calculate its mixing efficiency and pressure drop at Re = 1, 10, 25, 50, and 100. Figure 11 shows the variation of mixing efficiency and pressure drop of RD with increasing Re. Through research, it is found that at different Res, the pressure drop at the outlet of RD increases as the mixing efficiency increases. Their relationship is proportional. We are looking for a micromixer with high mixing efficiency and low pressure drop, so this means that the two objective functions of mixing efficiency and pressure drop are in a competitive relationship at any Res. As long as the objective functions are in competition, they can be optimized using the Pareto genetic algorithm, so the multi-objective optimization approach is applicable to different Res.

## Conclusion

In order to optimize the structure of the micromixer with Cantor fractal baffle through the multi-objective genetic algorithm, we select three design variables, and use the mixing index and pressure drop at the outlet of the micromixer as two objective functions for optimization. LHS is used to randomly sample to form a data set with 30 design points, and the RSA function is used to build a proxy modeling. Finally, we use the Pareto genetic algorithm to optimize the proxy modeling to obtain the Pareto optimal frontier. K-means clustering is used to classify the optimal solution set, and the representative design variables are selected as the research object. The following conclusions can be drawn through the research:

1. On OPT-B, compared with OPT-1, the mixing index of OPT-2 at the outlet is 8.1E-3 smaller than it, and the pressure drop is also 405.8153 Pa smaller than it. The results show that in the optimal solution set obtained by the Pareto genetic algorithm, we can weigh the value of the objective function for each set of solutions. Thus, a micromixer with excellent mixing performance and strong safety is obtained.
2. On OPT-A, as the mixing length increases, the mixing efficiency of the optimized micromixer is gradually higher than the RD. At the outlet of the micromixer, the mixing efficiency is increased by 20.59% at the maximum and 0.89% at the minimum compared to the RD.
3. On OPT-B, as the mixing length increases, the mixing efficiency of the optimized micromixer is gradually higher than the RD. At the outlet of the micromixer, the mixing efficiency is increased by 14.07% at the maximum and 5.55% at the minimum compared to the RD.
4. Compared with OPT-A, the pressure drop at the outlet of the micromixer on OPT-B is much larger, while the highest is 1411.91 Pa and the lowest is 485.91 Pa. It shows that when Re increases, the fluid in the micromixer enhances chaotic convection, which will increase the pressure drop of the micromixer.

Through the results of the study, it is found that the combination of fractal theory and multi-objective genetic algorithm can indeed improve the mixing performance of the micromixer. We can use the genetic algorithm to find the Pareto optimal frontier, that is, the set that indicates the trade-off between the objective functions can be obtained. Further, we can find the appropriate design parameters of the micromixer from the optimal set of solutions. In summary, the multi-objective optimization in this paper can make the micromixer as efficient as possible while keeping the pressure drop as low as possible, thus ensuring that the micromixer has a strong safety feature. Since the multi-objective optimization makes the microfluidic device both efficient and safe, it will bring more prospects to many fields such as microelectromechanical systems, chemical synthesis, and industrial systems.

## Data Availability

The data that support the findings of this study are available from the corresponding author upon reasonable request.
